# Supplementary material for: Stabilization of Silver Nanoparticles by Cationic Aminoethyl Methacrylate Copolymers in Aqueous Media—Effects of Component Ratios and Molar Masses of Copolymers
Source: Polymers (Basel). 2019 Oct 10;11(10):1647. doi: 10.3390/polym11101647 (PMC6835539; doi:10.3390/polym11101647)
Supplement: Supplementary file 1 [file polymers-11-01647-s001.pdf]

Supplementary Material.

Article

Stabilization of silver nanoparticles by cationic aminoethyl methacrylate copolymers in aqueous media: Effects of component ratios and molar masses of copolymers

Mariya E. Mikhailova [1], Anna S. Senchukova [1], Alexey A. Lezov [1], Alexander S. Gubarev [1], Anne -K. Trützschler [2,3], Ulrich S. Schubert [2,3] and Nikolay V. Tsvetkov [1,*]

[1] Department of Molecular Biophysics and Polymer Physics, St. Petersburg State University, St. Petersburg, 199034 Russia
[2] Laboratory of Organic and Macromolecular Chemistry (IOMC), Friedrich Schiller University Jena, Humboldtstr. 10, 07743 Jena, Germany
[3] Jena Center for Soft Matter (JCSM), Friedrich Schiller University Jena, Philosophenweg 7, 07743 Jena, Germany
* Correspondence: N.V. Tsvetkov, Department of Molecular Biophysics and Polymer Physics, St. Petersburg State University, St. Petersburg, 199034, Russia; E-mail addresses: n.tsvetkov@spbu.ru, n.tsvetkov@mail.ru; Tel.: +7-812-428-7598

1. Absorption spectra and time dependences of Abs_{max} values for various variation components cases:

- *The effect of the fraction of reducing agent*

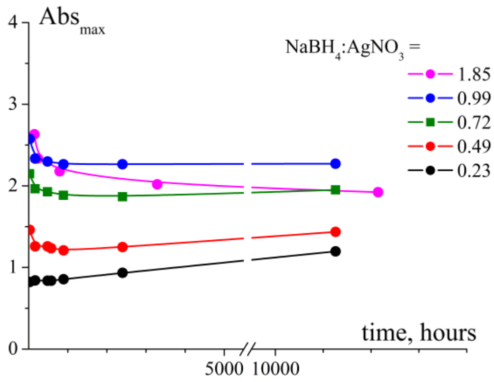

Figures S1. Time dependences of Abs_{max} values at various molar ratios between $NaBH_4$ and $AgNO_3$ are indicated in the image.

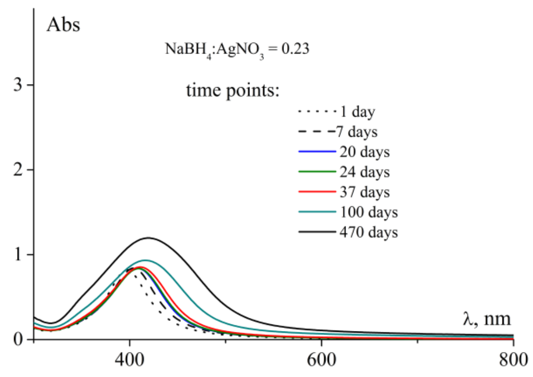

(a)

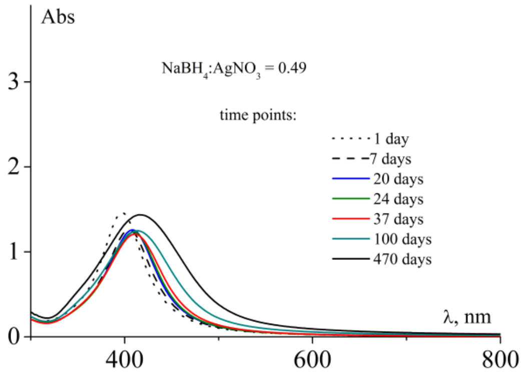

(b)

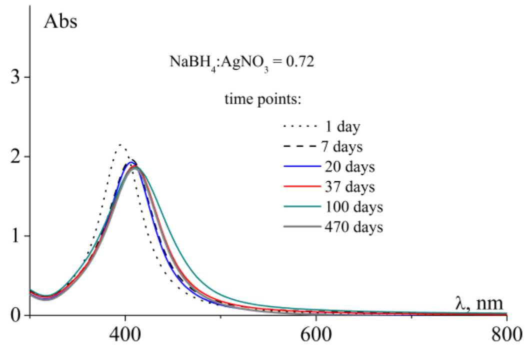

(c)

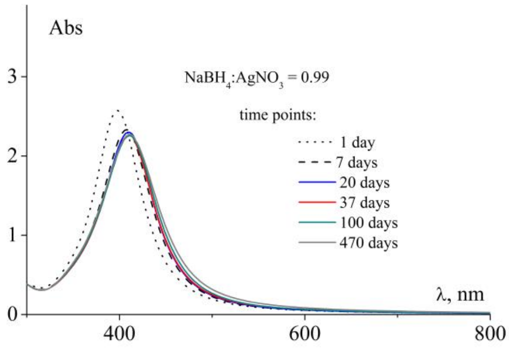

(d)

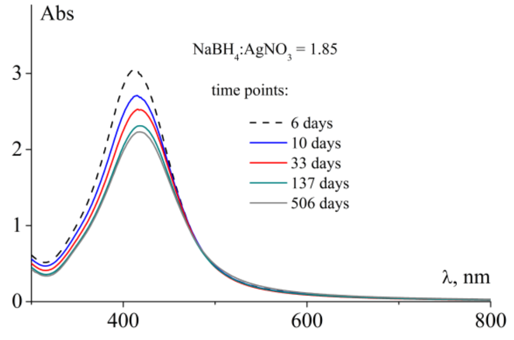

(e)

Figures S2. Absorption spectra taken in various point of time at various molar ratios between $NaBH_4$ and $AgNO_3$ are indicated in the image.

- *Holding capacity of the copolymers*

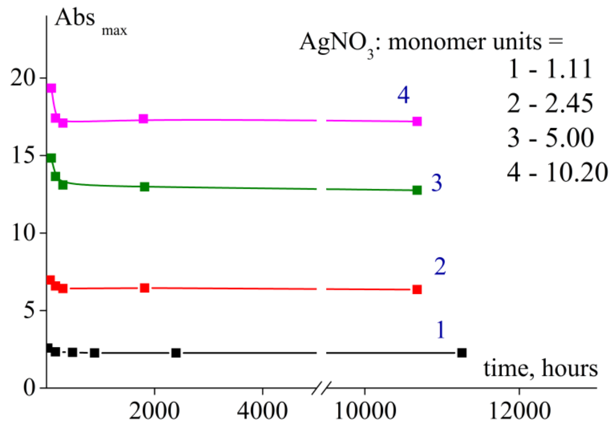

Figures S3. Time dependences of Absmax values at various molar ratios between $AgNO_3$ and monomer units are indicated in the image.

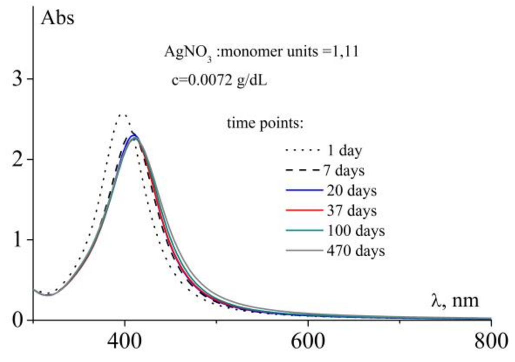

(a)

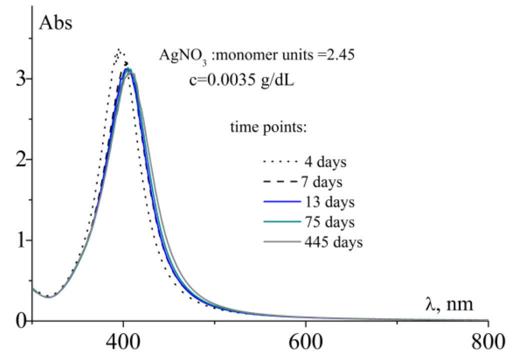

(b)

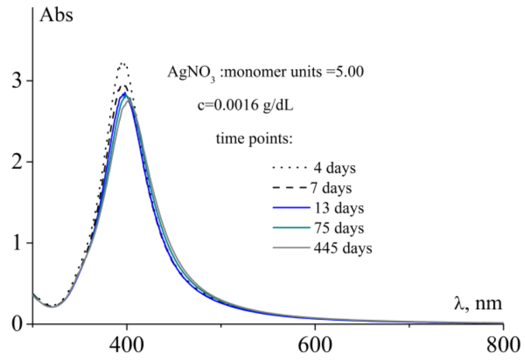

(c)

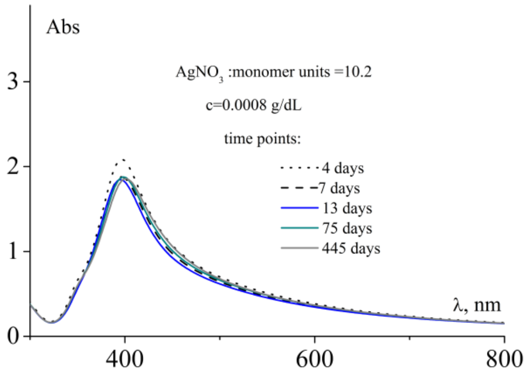

(d)

Figures S4. Absorption spectra taken in various point of time at various molar ratios between $AgNO_3$ and monomer units are indicated in the image.

3. Steps of regularization methods procedure (for examples):

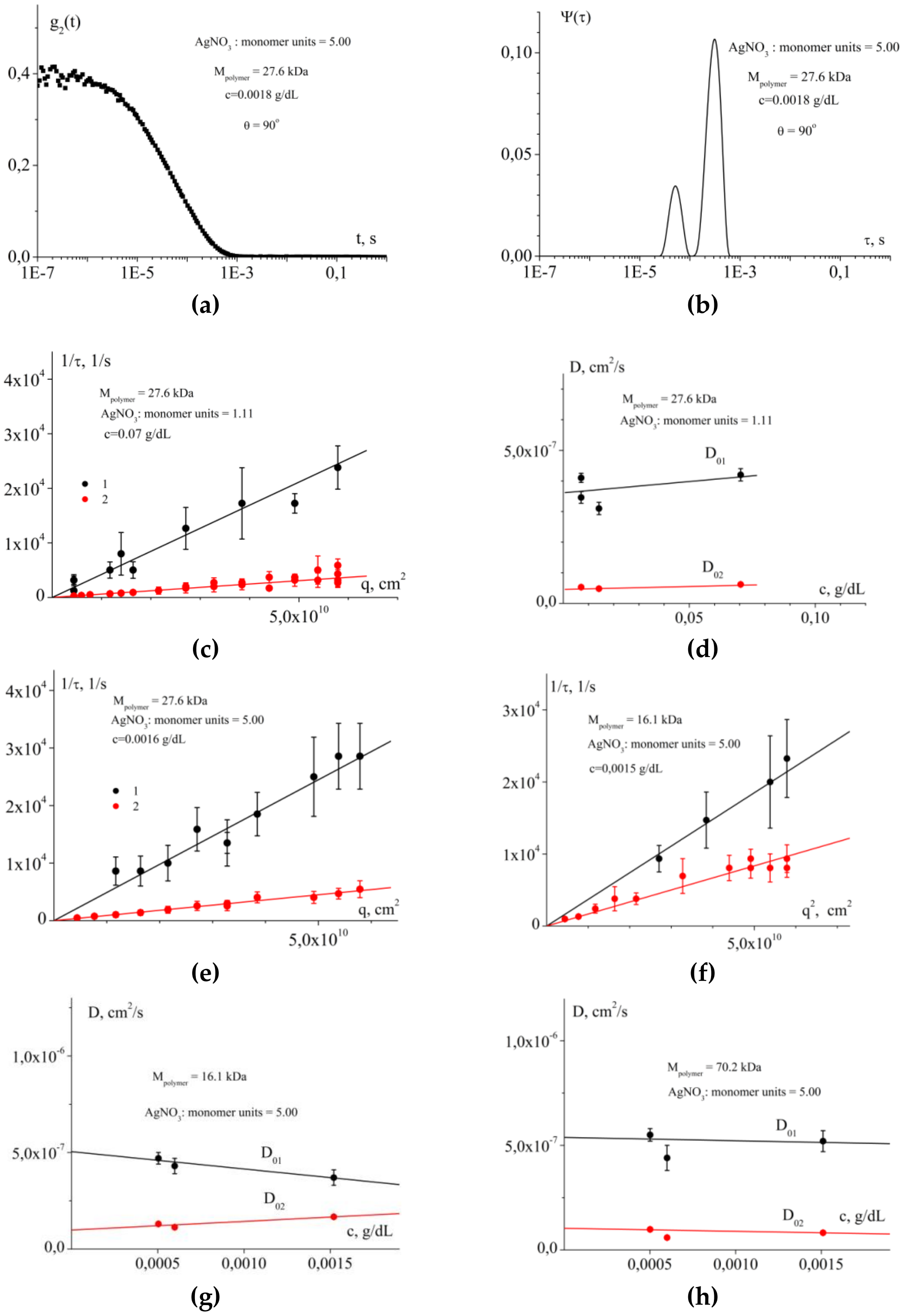

Figures S5. (**a**) Normalized intensity homodyne autocorrelation function; (**b**) Distribution of relaxation times; (**c**), (**e**), (**f**) Inverse relaxation time 1/τ on scattering vector squared q^2; (**d**), (**g**), (**h**) Concentration dependences of translation diffusion coeffitients. $M_{polymer}$, concentrations, molar ratios ($AgNO_3$: monomer units) and scattering angle is indicated in the images.

4. SEM images of polymer/NP samples (for examples):

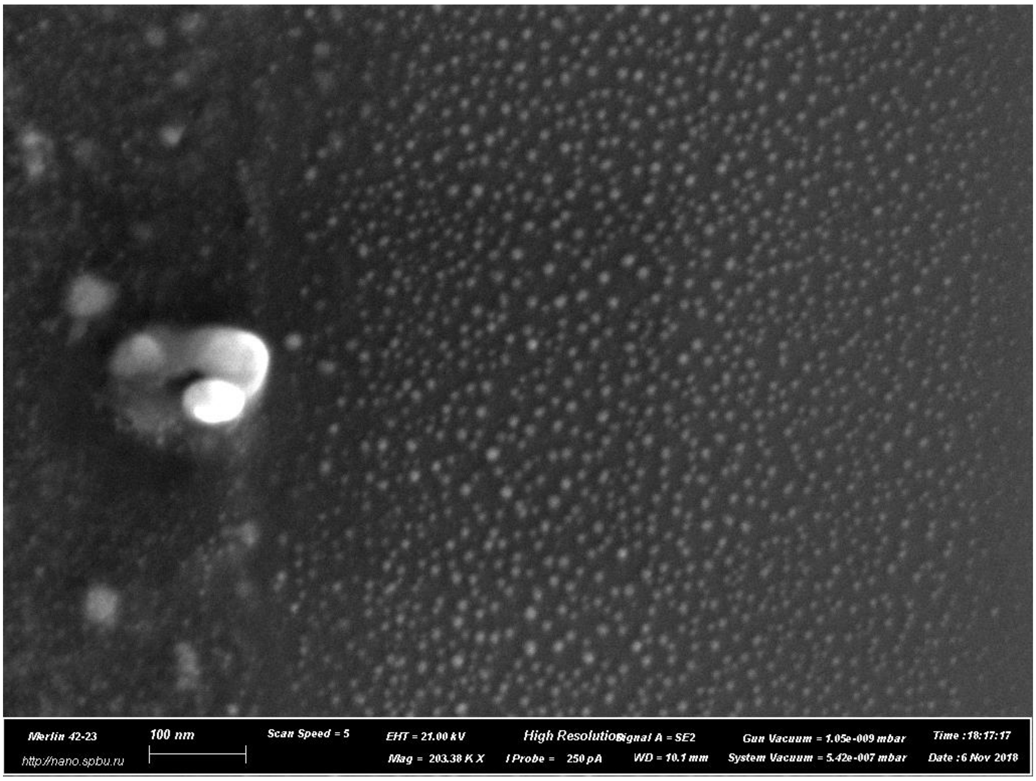

(a) $M_{polymer}$ = 27.6 kDa, ($NaBH_4$:$AgNO_3$) = 0.23, ($AgNO_3$:$NaBH_4$)=1.14.

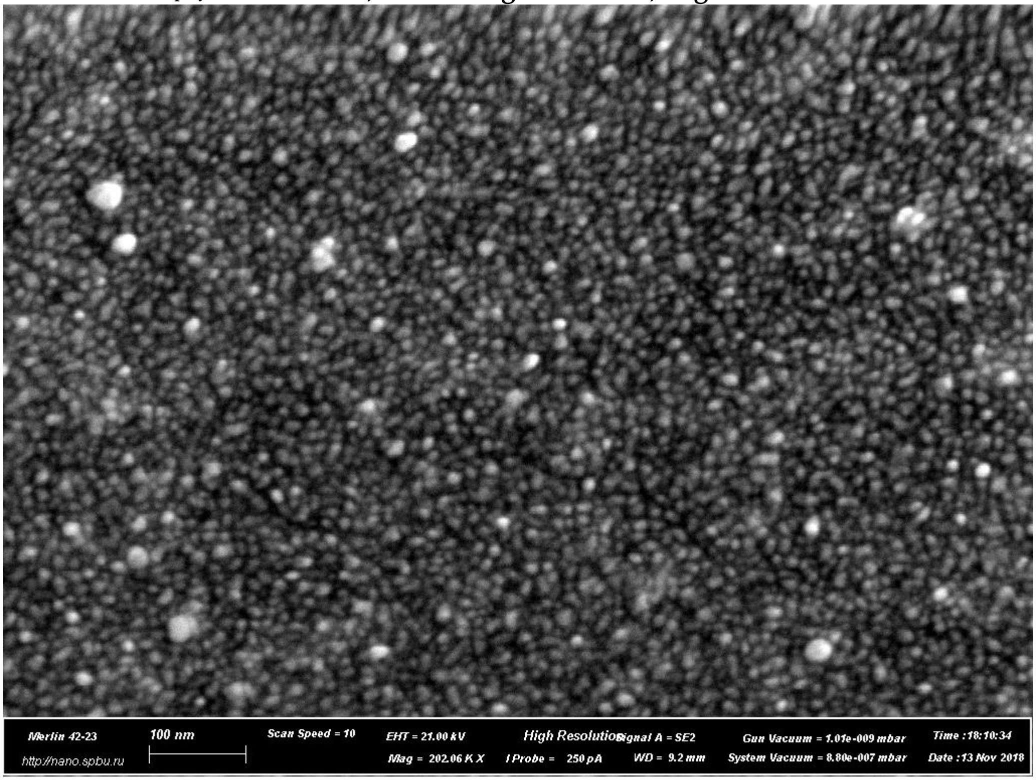

(b) $M_{polymer}$ = 27.6 kDa, ($NaBH_4$:$AgNO_3$) = 0.99, ($AgNO_3$:$NaBH_4$)=1.14.

Figures S6. SEM images of polymer/NP samples. $M_{polymer}$ and molar ratios is indicated in the images.

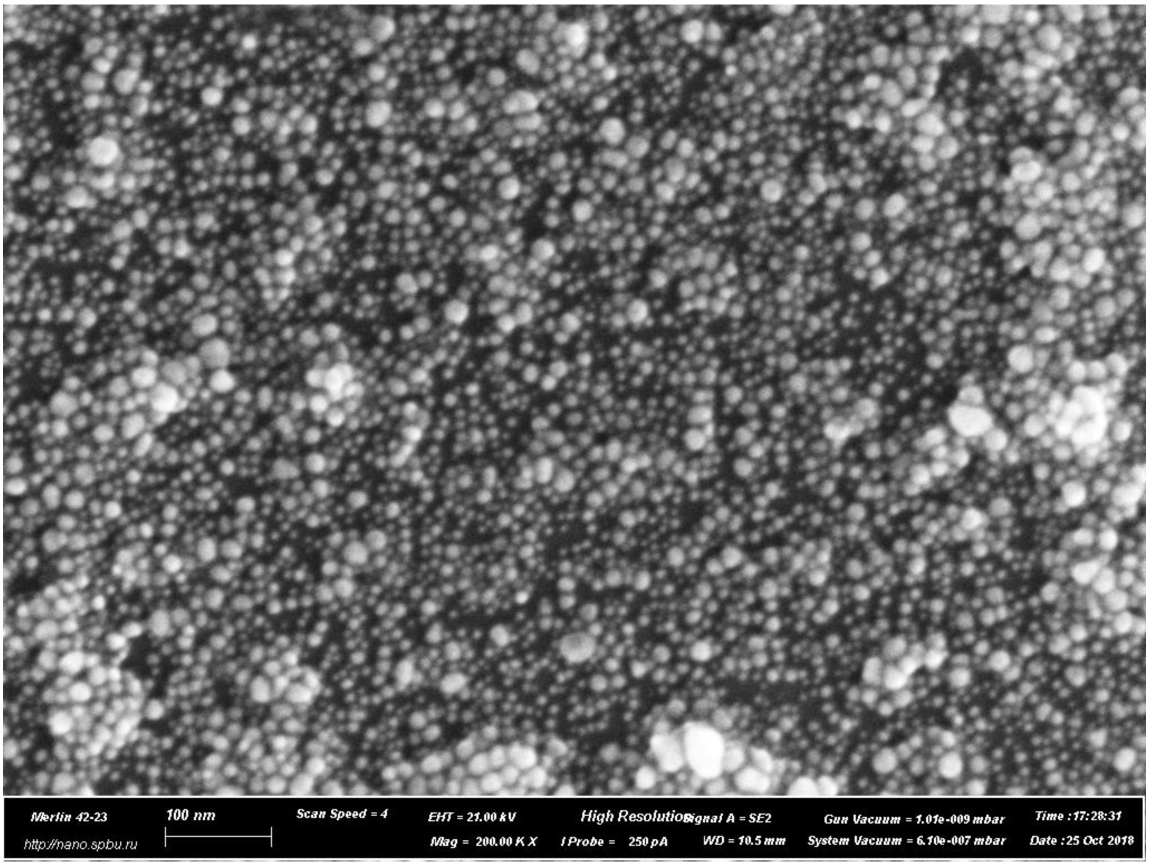

(c) $M_{polymer}$ = 27.6 kDa, ($NaBH_4$:$AgNO_3$) = 0.96, ($AgNO_3$:$NaBH_4$)=5.00.

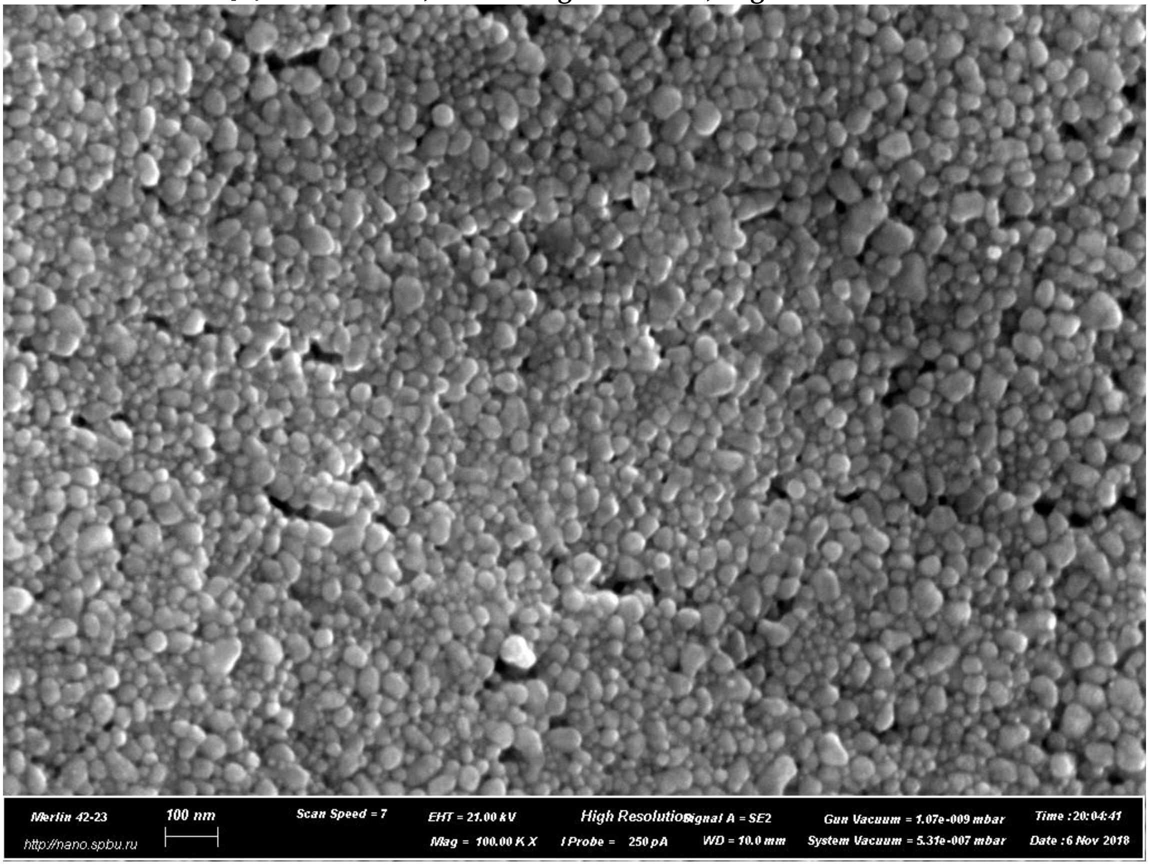

(d) $M_{polymer}$ = 27.6 kDa, ($NaBH_4$:$AgNO_3$) = 0.96, ($AgNO_3$:$NaBH_4$)=10.2.

Figures S6. SEM images of polymer/NP samples. $M_{polymer}$ and molar ratios is indicated in the images.
